# Comprehensive analysis of the complete mitochondrial genome of *Distylium chinense*

**DOI:** 10.3389/fpls.2026.1820003

**Published:** 2026-05-15

**Authors:** Bo Ding, Yan-bin Xue, Chang-ying Xia, Zhi-wei Huang, Zi-yun Shu, Hua Wang

**Affiliations:** 1 College of Agriculture and Forestry Science and Technology, Chongqing Three Gorges Vocational College, Chongqing, China; 2 Design College, Chongqing Industry Polytechnic University, Chongqing, China; 3 School of Life Sciences, Southwest University, Chongqing, China; 4 Chongqing Academy of Chinese Materia Medica, Chongqing, China; 5 College of Teacher Education, Southwest University, Chongqing, China; 6 State-owned Baima Mountain Forest Farm, Chongqing, China

**Keywords:** *Distylium chinense*, evolution analysis, mitochondrial genome, repeat sequence, RNA editing

## Abstract

**Introduction:**

*Distylium chinense* is a flood-tolerant woody species distributed in the hydro-fluctuation zone of the Three Gorges Reservoir in the Yangtze River basin and plays an important role in regional ecological restoration. Nevertheless, its mitochondrial genome (mitogenome) has not yet been characterized.

**Methods:**

Here, the complete mitogenome of *D. chinense* was de novo assembled using a hybrid sequencing strategy integrating MGISEQ-2000 and Nanopore platforms. Codon usage bias was evaluated based on relative synonymous codon usage (RSCU). Simple sequence repeats (SSRs), mitochondrial plastid DNA (MTPTs) fragments, and RNA editing sites were also identified.

**Results:**

The assembled mitogenome was 916,504 bp in length, with a GC content of 46.17%, and contains 39 unique protein-coding genes (PCGs), 19 transfer RNA (tRNA) genes, and 3 ribosomal RNA (rRNA) genes. A total of 294 SSRs, 17 MTPTs fragments, and 676 RNA editing sites were detected in the mitogenome. Phylogenetic analysis placed *D. chinense* within the order Saxifragales, showing the closest relationship to *Distylium racemosum*.

**Discussion:**

Collectively, these results provide a comprehensive characterization of the *D. chinense* mitogenome and establish a valuable genomic resource for future studies on mitochondrial genome evolution and the molecular basis of flood tolerance.

## 1 Introduction

Flooding is a widespread abiotic stress resulting from both natural processes (e.g., heavy rainfall and snowmelt) and anthropogenic activities such as hydropower development, which alter hydrological regimes. In the Three Gorges Reservoir region, seasonal water level fluctuations induced by dam operation impose prolonged and recurrent flooding stress, leading to hypoxia, oxidative stress, and metabolic imbalance in plants (Duan, 2019). Therefore, elucidating the mechanisms underlying plant flooding tolerance and identifying suitable species for ecological restoration are critical for maintaining ecosystem stability and ensuring the long-term sustainability of the reservoir region. *Distylium chinense* (Hamamelidaceae) is an endangered evergreen shrub naturally distributed in the hydro-fluctuation zone of the Three Gorges Reservoir area (Zhang et al., 2003). It exhibits remarkable flood tolerance, largely attributed to its well-developed and intertwined root system, which enables survival under prolonged submergence of up to six months (Chen et al., 2008). Consequently, *D. chinense* has been widely recognized as a key species for riparian vegetation restoration, soil stabilization, and bank protection (Li et al., 2011a; Liu et al., 2014; Sun et al., 2020), and also holds ornamental value (Xiang et al., 2020). However, the construction and operation of the Three Gorges Dam have resulted in extensive habitat submergence, leading to significant declines in natural populations of *D. chinense*. Combined with anthropogenic disturbances such as overharvesting, both population size and genetic diversity have been severely reduced, causing the gradual loss of natural communities (Sun et al., 2020). Despite its ecological importance, the molecular basis underlying its flood tolerance remains poorly understood. Therefore, comprehensive genomic characterization of *D. chinense* is essential for both conservation and functional studies.

Mitochondria are essential organelles responsible for cellular energy metabolism and play central roles in plant growth, development, and stress responses (Møller et al., 2021). Plant mitochondrial genomes (mitogenomes) are characterized by large size variation, complex structures, conserved gene content, extensive non-coding regions, and frequent RNA editing events. In angiosperms, mitogenome sizes typically range from 200 kb to 3 Mb. Despite the rapid accumulation of plastid genome data, plant mitogenomes remain underrepresented. To date, nearly 13,000 plastid genomes have been reported in public databases, whereas only a limited number of complete plant mitogenomes are available, covering relatively few species (Wang et al., 2024). Recent advances in long-read sequencing technologies, particularly Nanopore sequencing, have greatly facilitated the assembly of complex organellar genomes.

In the present study, we assembled the complete mitogenome of *D. chinense* using a hybrid sequencing strategy combining MGISEQ-2000 and Nanopore technologies. We systematically characterized its genomic structure, gene content, codon usage patterns, and RNA editing events. In addition, mitochondrial plastid DNA (MTPT) transfer events and phylogenetic relationships were analyzed, and comparative mitogenomic analyses were conducted with related species. The objectives of this study were to: (1) conduct a comprehensive analysis of the mitochondrial genome architecture and sequence divergence of *D. chinense*; (2) examine gene transfer events between its chloroplast and mitochondria; (3) investigate organellar genome evolution and the molecular mechanisms associated with flood tolerance in this species.

## 2 Materials and methods

### 2.1 Plant material, DNA extraction, and sequencing

Fresh leaves of *D. chinense* were collected from a greenhouse at Southwest University (Chongqing, China). The samples were thoroughly rinsed with ultrapure water before being stored at -80°C for subsequent use. Total genomic DNA was isolated using a modified cetyltrimethylammonium bromide (CTAB) method (Adamovic et al., 2014) and further purified using a commercial plant genomic DNA extraction kit (Tiangen Biotech Co., Ltd., Beijing, China). DNA quality and concentration were assessed using a NanoDrop 2000 spectrophotometer (Thermo Fisher Scientific, USA) and 1.0% agarose gel electrophoresis. Whole-genome sequencing was performed by Beijing Genomics Institute (BGI) using both MGISEQ-2000 and Nanopore platforms.

### 2.2 Assembly and annotation of mitogenome

The *D. chinense* mitochondrial genome was *de novo* assembled from long-read sequencing data according to the previously described pipeline (Kolmogorov et al., 2019) with default parameters, and an initial assembly graph was generated in GFA format. Subsequently, assembled contigs in FASTA format were imported and indexed by constructing a local BLAST database. A nucleotide BLAST search was then performed to screen and extract mitochondrial-specific sequences. For assembly quality validation, long reads were further applied to confirm and verify the structural connections among independent contigs. The mitogenome of the closely related species *Distylium racemosum* (accession numbers PQ594873.1–PQ594874.1) was employed as the reference, with the following parameters: -evalue 1e-5 -outfmt 6 -max_hsps 10 -word_size 7 -task blastn-short. The assembly graph was visualized using Bandage (Wick et al., 2015), and mitochondrial contigs were manually curated and extracted to generate the final mitogenome assembly. Genome annotation was performed using the PMGA pipeline (Li et al., 2024) with a reference database comprising 319 plant mitochondrial genomes. Transfer RNA (tRNA) genes were predicted using tRNAscan-SE (v 2.0.11) (Lowe and Eddy, 1997), while ribosomal RNA (rRNA) genes were identified using BLASTn (v 2.16.0) (Chen et al., 2015). Any annotation errors detected were manually corrected using Apollo software (version 1.11.8) (Lewis, et al., 2002). Finally, the complete mitochondrial genome map was visualized with OGDRAW software (Stephan et al., 2019; Quang et al., 2020).

### 2.3 Analysis of codon usage and repeated sequences

Protein-coding gene (PCG) sequences were extracted following Zhang et al. (2020). Codon usage bias was evaluated using MEGA (v 7.0) (Kumar et al., 2016), and relative synonymous codon usage (RSCU) values were calculated. Simple sequence repeats (SSRs) were identified using MISA (Beier et al., 2017), while tandem repeats were detected using Tandem Repeats Finder (TRF v 4.09) (Benson, 1999). Dispersed repeats were identified using Repeat Masker (Wynn and Christensen, 2019). The results were visualized using Microsoft Excel 2010 and Circos (v 0.69.9) (Zhang et al., 2013).

### 2.4 Identification of MTPTs, and RNA editing events

The chloroplast genome was assembled from short-read data using GetOrganelle (v 1.7.7.1) (Jin et al., 2020) and annotated using CPGAVAS2 (Shi et al., 2019), followed by manual refinement with CPGView (Liu et al., 2023). Homologous sequences between mitochondrial and chloroplast genomes were identified using BLASTn (v 2.16.0) and visualized using Circos (v 0.69.9). These homologous fragments were defined as mitochondrial plastid DNA (MTPT) sequences. All mitochondrial PCGs were used as input to predict RNA editing sites. C-to-U RNA editing events were predicted using Deepred-mt (Edera et al., 2021) under default parameters.

### 2.5 Phylogenetic and synteny analyses

Closely related species were selected based on phylogenetic relevance, and their mitochondrial genome sequences were retrieved from public databases. Shared PCGs were extracted using PhyloSuite (v 1.1.16) (Zhang et al., 2020). Genes were selected according to their presence across all 34 analyzed species. Genes missing in any taxon were excluded from phylogenetic analysis. In total, 17 conserved protein-coding genes shared by all sampled species were retained. Based on the Angiosperm Phylogeny Group IV (APG IV) classification system, outgroup taxa were chosen from families phylogenetically closest to the ingroup. Multiple sequence alignment was performed using MAFFT (v 7.525) (Katoh and Standley, 2013). A maximum likelihood (ML) phylogenetic tree was constructed using IQ-TREE (v 2.3.6) (Minh et al., 2020) with 1,000 ultrafast bootstrap replicates and 1,000 SH-aLRT tests. The resulting tree was visualized using iTOL (v 7) (Letunic and Bork, 2024). Syntenic blocks were identified using BLASTn, and only homologous regions longer than 500 bp were retained. Pairwise synteny analyses were conducted using MCScanX (Wang et al., 2012) to identify conserved collinear regions, which were subsequently visualized.

## 3 Results

### 3.1 General features of *D. chinense* mitogenome

**Figure 1:**
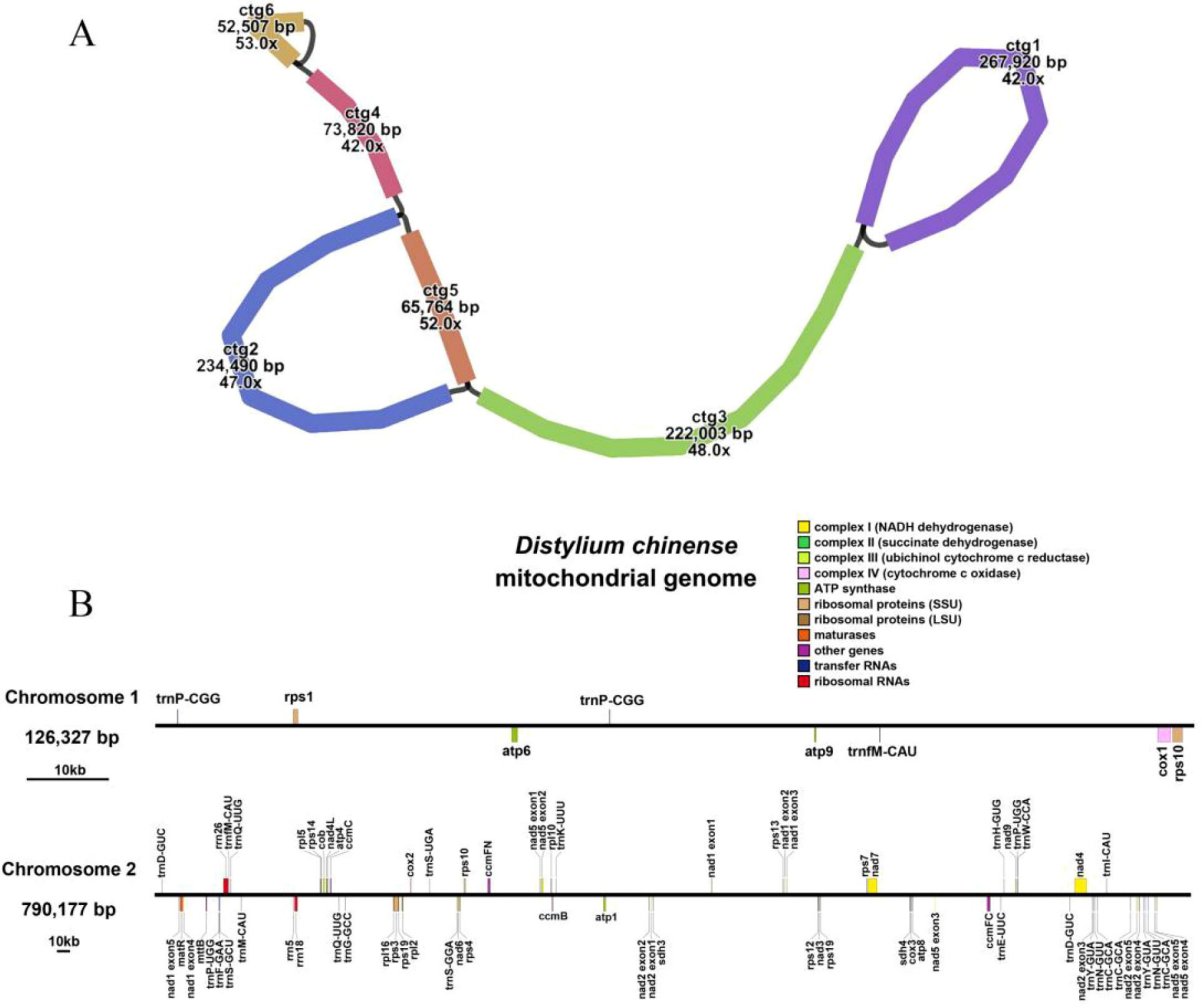
Mitogenomic map of *D. chinense*. **(A)** Circular map of the mitochondrial genome showing six contigs (ctg1–ctg6) with base pair sizes and coverage values. **(B)** Linear maps of two chromosomes (126,327 bp and 790,177 bp) with annotated gene names and color-coded gene types.

*De novo* assembly was performed on all HiFi sequencing data using the Flye software. In total, the assembly yielded 417,533,106 bp, comprising 6,083 contigs. The longest contig was 471,474 bp, and the contig N50 was 91,137 bp. Following homology identification, six contigs homologous to mitochondrial gene sequences were identified from the 6,083 contigs. These six sequences contained known mitochondrially encoded genes and were interconnected with one another, exhibiting no breakpoints or gaps. The mitochondrial genome assembly of *D. chinense* consisted of 6 contigs that were interconnected to form 8 linkages, as illustrated in **Figure 1A**. Specifically, ctg6 could form a self-circularized structure (corresponding to ctg6(+) > ctg6(+)) and could also be linked to ctg4 (ctg4(+) > ctg6(−)). Similarly, ctg1 could self-circularize (ctg1(+) > ctg1(+)) or connect with ctg3 (ctg1(+) > ctg3(−)). Ctg2 could be joined to ctg4 (ctg4(−) > ctg2(+)) or form a circular structure with ctg5 via two linkages: ctg2(+) > ctg5(+) and ctg5(+) > ctg2(+).Finally, ctg5 could connect to ctg3 (ctg5(−) > ctg3(+). The mitogenome of *D. chinense* exhibited a highly complex, multibranched, multichromosomal structure. It had a total length of 916,504 bp and a GC content of 46.17%. Genome annotation identified 39 unique protein-coding genes (PCGs), including two multicopy genes, comprising 24 core mitochondrial genes and 15 non-core genes. In addition, 19 transfer RNA (tRNA) genes (eight multicopy) and 3 ribosomal RNA (rRNA) genes were identified (Genbank: contig1 PZ247528, contig2 PZ247529) (**Figure 1B**). Among the core genes, five encode ATP synthase subunits (*atp*1, *atp*4, *atp*6, *atp*8, and *atp*9), nine encode NADH dehydrogenase subunits (*nad*1, *nad*2, *nad*3, *nad*4, *nad4L*, *nad*5, *nad*6, *nad*7, and *nad*9), and four were involved in cytochrome C biogenesis (*ccmB*, *ccmC*, *ccmFC*, and *ccmFN*). Additionally, three genes encode cytochrome C oxidase subunits (*cox*1, *cox*2, and *cox*3), while *mttB* encodes a membrane transport protein, *matR* encodes a maturase, and *cob* encodes cytochrome b. The non-core genes included four large ribosomal subunit genes (*rpl*2, *rpl*5, *rpl*10, *rpl*16) and nine small ribosomal subunit genes (*rps*1, *rps*3, *rps*4, *rps*7, *rps*10, *rps*12, *rps*13, *rps*14, *rps*19), as well as two succinate dehydrogenase genes (*sdh*3 and *sdh*4).

In addition, we also produced the complete *D. chinense* chloroplast genome (Genbank: PZ247530). This genome was subsequently used for comparative analysis with the *D. chinense* mitochondrial genome in order to identify the MTPTs.

### 3.2 Analysis of codon usage in PCGs

**Figure 2:**
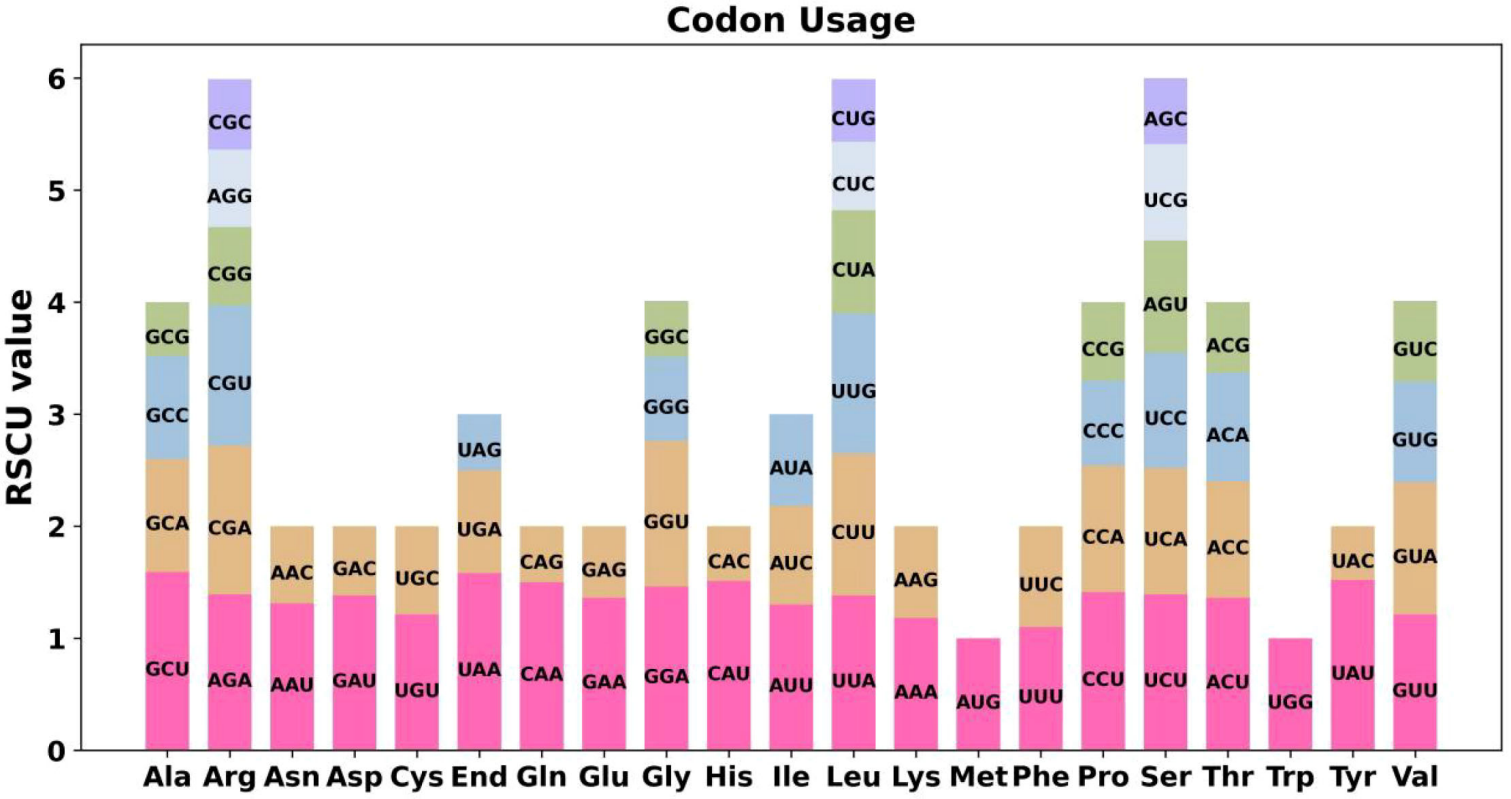
Codon usage patterns of 20 amino acids and stop codons among all PCGs in the *D. chinense* mitogenome. Different colors are used to represent different codons in the histogram.

Codon usage bias in the protein-coding genes (PCGs) of the *D. chinense* mitogenome was systematically analyzed. Codon usage frequencies for each amino acid are summarized in **Supplementary Table 1**. Codons with relative synonymous codon usage (RSCU) values greater than 1 were considered preferentially used. As shown in **Figure 2**, pronounced codon usage bias was observed across mitochondrial PCGs. Notably, the start codon AUG and tryptophan (Trp, UGG), exhibited no bias, each with an RSCU value of 1. Among all codons, alanine (Ala) showed a strong preference for GCU, which exhibited the highest RSCU value (1.59), indicating a significant codon usage bias in the *D. chinense* mitogenome.

### 3.3 Investigation of repeat sequences and recombination events

**Figure 3:**
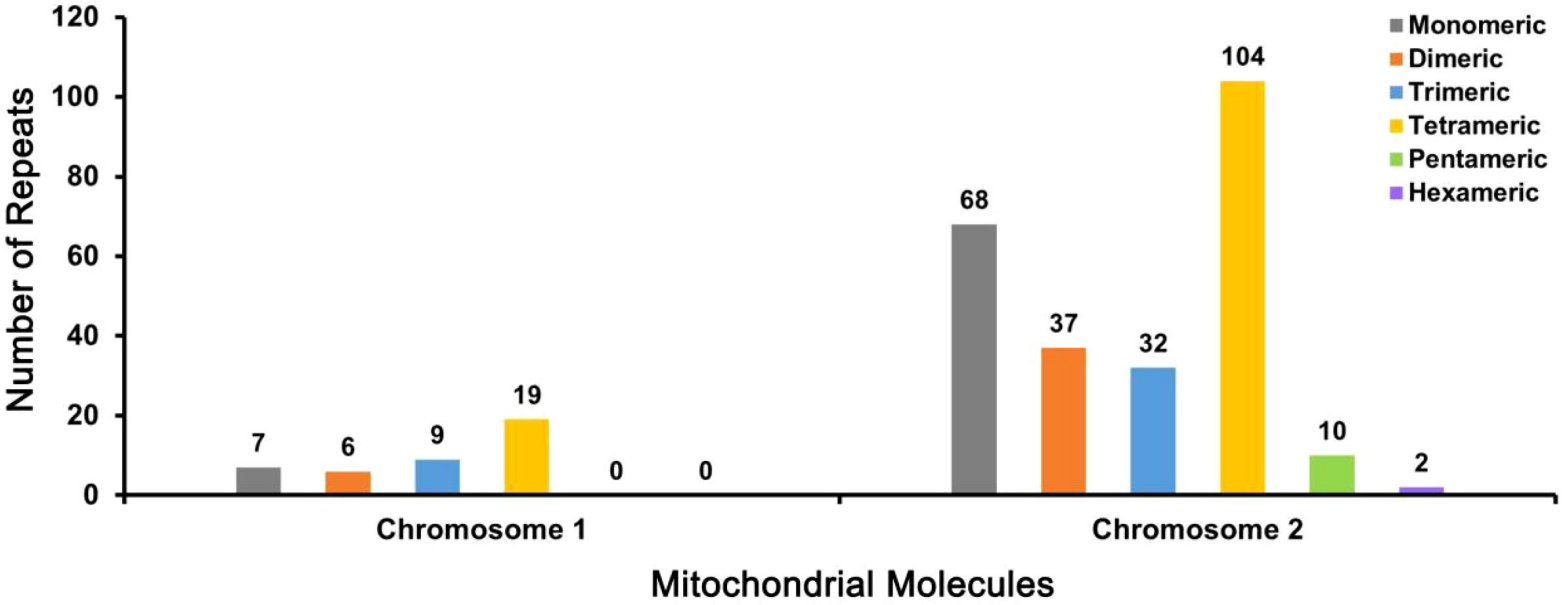
Distribution of SSRs on Chromosome 1 and Chromosome 2 in the mitogenome of *D. chinense*. The SSRs were color-coded as follows: monomeric (gray), dimeric (orange), trimeric (blue), tetrameric (yellow), pentameric (green), and hexameric (purple).

A large number of repetitive sequences were identified in the *D. chinense* mitogenome. In total, 294 simple sequence repeats (SSRs) were detected, with 41distributed on Chromosome 1 and 253 on Chromosome 2 (**Figure 3**). Among these SSRs, tetrameric motifs were the most abundant, accounting for 41.84% of the total. Of the 75 monomeric SSRs, thymine (T) repeats were predominant, comprising 52.00% (39 repeats). In addition, 54 tandem repeats were identified, with sequence identities exceeding 79% and lengths ranging from 7 to 71 bp (**Supplementary Table 2**). Of these, 6 were located on Chromosome 1 and 48 on Chromosome 2. Furthermore, analysis of interspersed repeats identified 1,054 repeat pairs longer than 30 bp, including 520 palindromic repeats and 534 forward repeats. The largest repeat unit reached 13,641 bp in length (**Supplementary Table 3**).

### 3.4 RNA editing events

**Figure 4:**
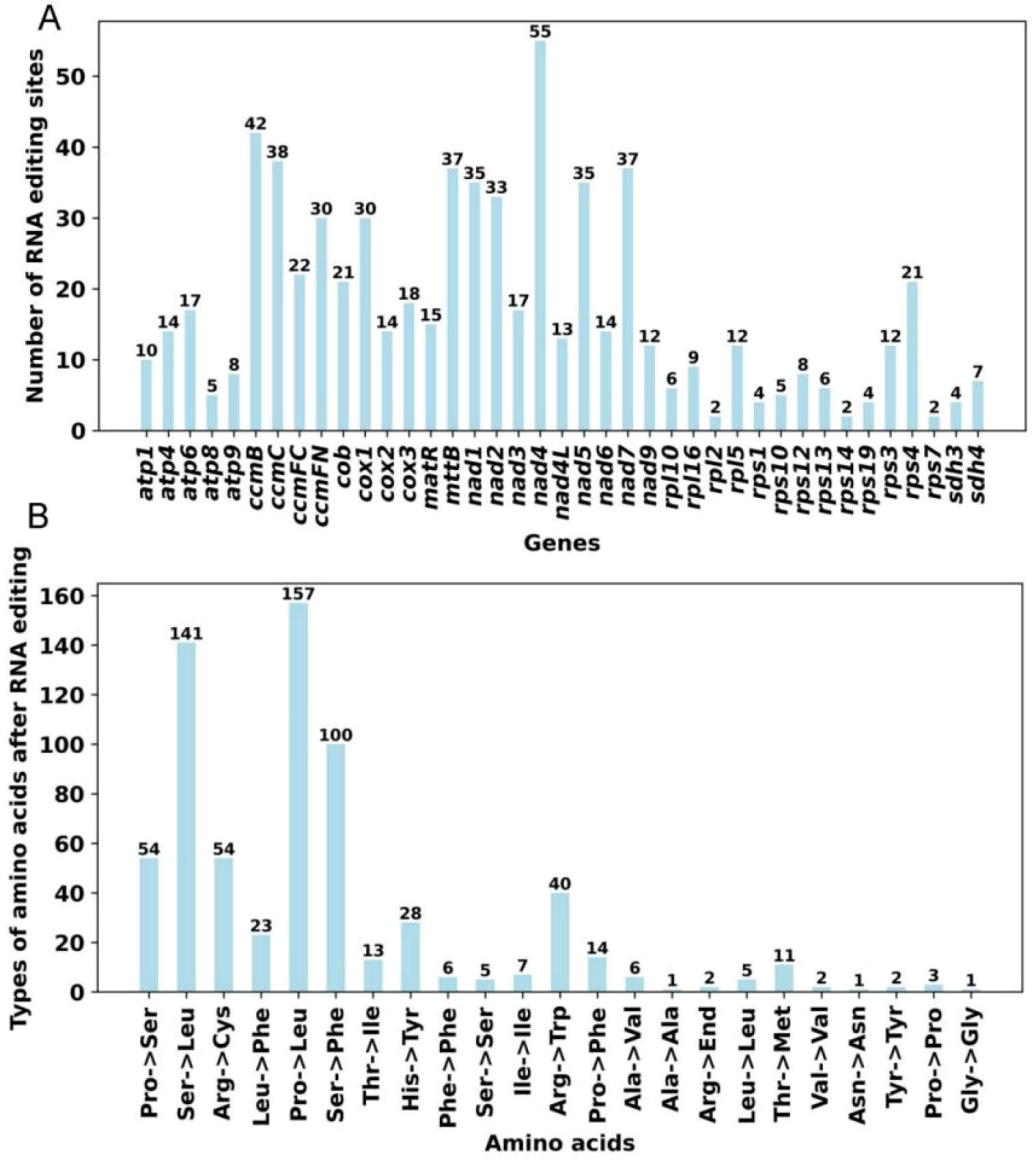
Predicted RNA editing sites **(A)** and corresponding amino acid changes **(B)** in *D. chinense* mitogenome PCGs.

RNA editing was a crucial mechanism for post-transcriptional modification in plant mitochondrial genomes. To ensure high-confidence predictions, only editing sites with a probability greater than 0.9 were retained. Across the 39 PCGs, we predicted a total of 676 RNA editing events, all of which were cytidine-to-uridine (C-to-U) conversions. The *nad*4 gene contained the most editing sites (47), followed by *ccmB* with 42 sites (**Figure 4A**). These editing events resulted in both synonymous and nonsynonymous substitutions. Specifically, nine types of synonymous substitutions (e.g., Ala→Ala, Gly→Gly) and fourteen types of nonsynonymous substitutions (e.g., Ser to Leu, Pro to Ser) (**Figure 4B**). A key functional consequence of these edits was the creation of start and stop codons, which was observed in eleven genes. Specifically, stop codons occurred in *ccmFC* and *rps*10, while new start codons were present in *cox*1, *cox*2, *nad*1, *nad*4L, *nad*5, *nad*7, *rps*1, *rps*3, and *rps*10. Notably, the creation of start codons in *cox*2 and *nad*5 each required editing at two separate sites (**Supplementary Table 4**). The mitochondrial C-to-U RNA editing exerted profound effects on gene expression and protein function by generating start codons, regulating stop codons, and altering amino acid sequences. This post-transcriptional modification mechanism not only guaranteed the accurate expression of mitochondrial genes but also participated in diverse critical biological processes, including plant growth and development, environmental adaptation, and stress tolerance.

### 3.5 Phylogenetic and synteny analyses

**Figure 5:**
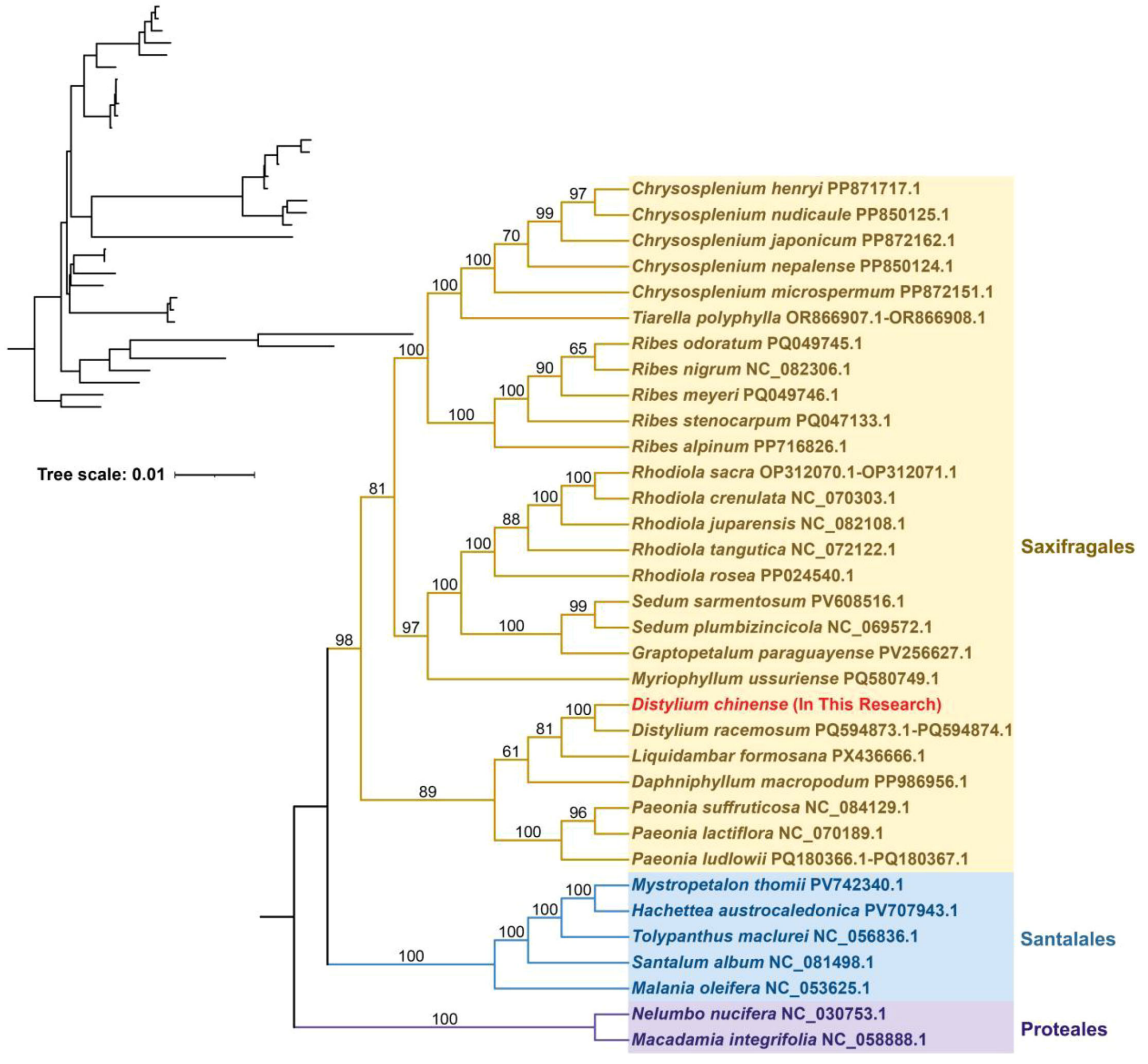
Phylogenetic tree of *D. chinense* and 34 additional plant species inferred from nucleotide sequences of 17 conserved core protein-coding genes in the mitogenome. *Nelumbo nucifera* and *Macadamia integrifolia* were used as outgroups. Bootstrap values are shown at each node.

To explore the evolutionary position of *D. chinense*, a phylogenetic tree was constructed based on the nucleotide sequences of 17 conserved core mitochondrial protein-coding genes (PCGs) (*atp*1, *atp*4, *atp*6, *ccmC*, *ccmFC*, *cox*1, *cox*2, *matR*, *nad*1, *nad*2, *nad*4L, *nad*5, *nad*6*, nad*7, *nad*9, *rpl*5, *rps*14) from 34 species representing three orders: Saxifragales, Santalales, and Proteales (**Figure 5**; **Supplementary Table 5**). *Nelumbo nucifera* and *Macadamia integrifolia* (Proteales) were selected as outgroups. In general, the topological structure of the mitochondrial genome-based phylogenetic tree accorded well with the most recent Angiosperm Phylogeny Group (APG) classification. Within the tree, *D. chinens*e was placed in the order Saxifragales and showed the closest evolutionary relationship with *Distylium racemosum* (**Figure 5**).

### 3.6 Characterization of MTPTs

**Figure 6:**
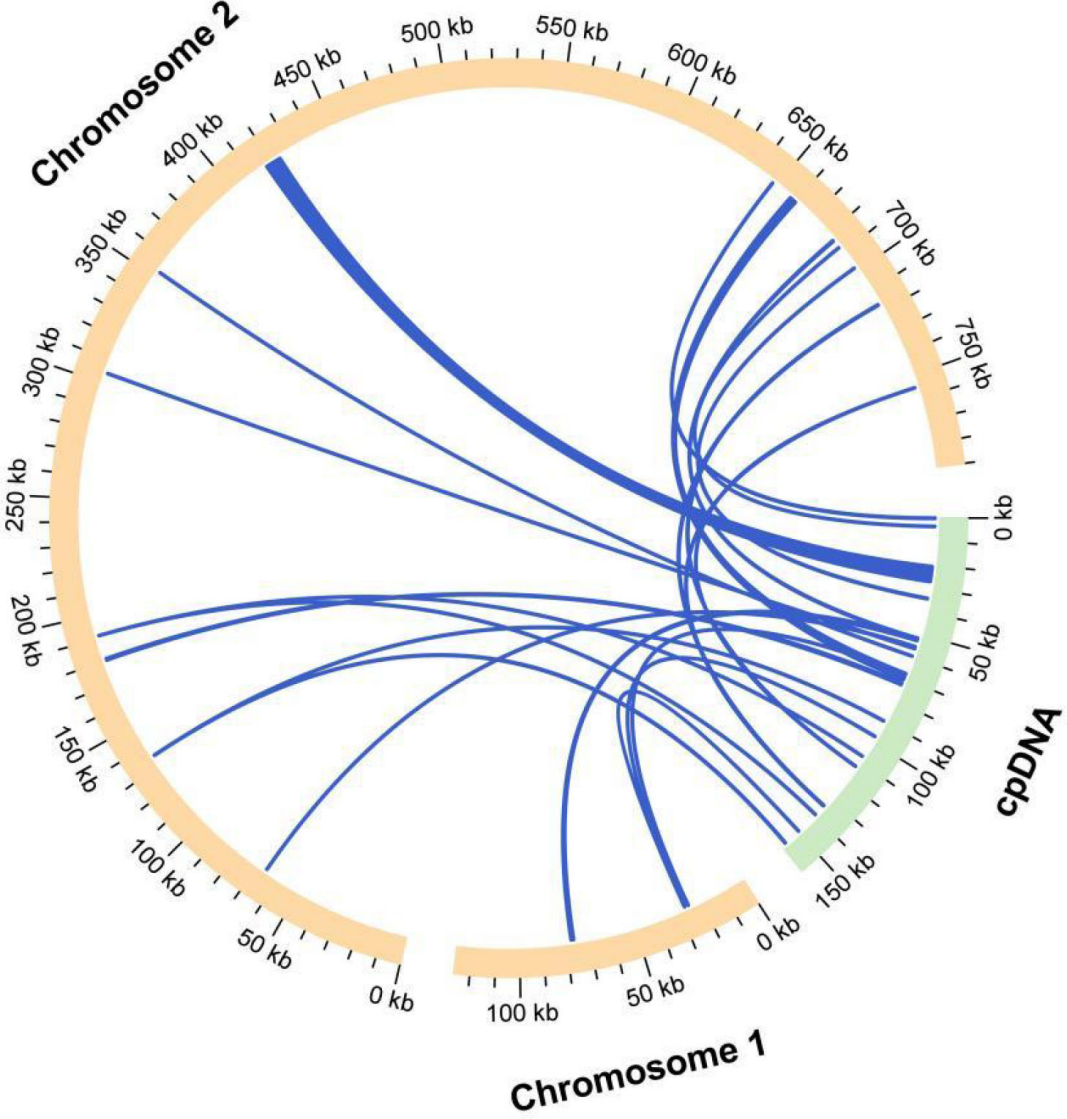
Analysis of homologous fragments between the chloroplast and mitochondrial genomes of *D. chinense*. The yellow and green arcs denote the mitochondrial genome and chloroplast genome, respectively. Homologous genomic regions are indicated by blue lines.

Based on sequence similarity analysis, a total of 17 homologous fragments were identified between the mitochondrial and chloroplast genomes of *D. chinense* (**Figure 6**). The cumulative length of these homologous regions was 11,981 bp, representing 1.31% of the complete mitochondrial genome. Among these fragments, three exceeded 1,000 bp in length and were derived from chloroplast protein-coding genes, transfer RNA (tRNA) genes, and intergenic regions (**Supplementary Table 6**). Among these transferred segments, MTPT5 was the largest one, with a length of 6,466 bp. By annotating these homologous sequences, 15 complete genes were further identified across 17 homologous fragments, including 9 protein-coding genes (*ndhJ*, *petG*, *petL*, *psbE*, *psbF*, *psbJ*, *psbL*, *rpl*20, *rpoC1*) and 6 tRNA genes (*trnH-GUG*, *trnI-CAU*, *trnM-CAU*, *trnN-GUU*, *trnP-UGG*, *trnW-CCA*).

**Figure 7:**
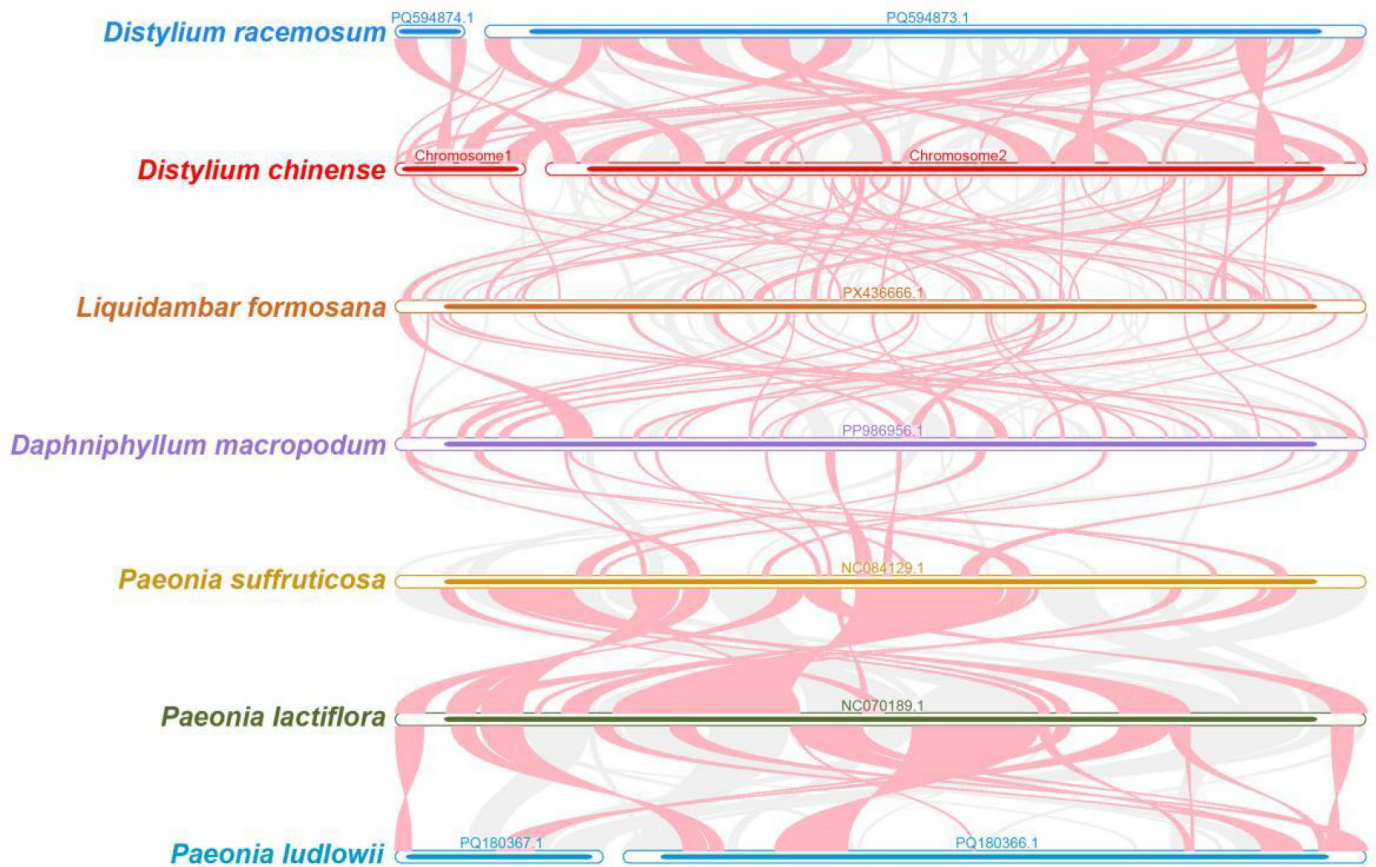
Synteny analysis of the mitochondrial genome in *D. chinense*. Red arcs represent inverted genomic regions, and gray blocks indicate highly homologous segments. Syntenic regions shorter than 0.5 kb were excluded and not displayed in the figure.

Based on sequence similarity analysis, comparative synteny analysis was performed between *D. chinense* and six related species. Multiple homologous syntenic blocks were identified, particularly between *D. chinense* and *D. racemosum* (**Figure 7**). Meanwhile, the arrangement of syntenic blocks varied among the seven mitochondrial genomes. In comparison with its close relatives, the mitogenome of *D. chinense* exhibited obvious genomic rearrangements, suggesting a low level of structural conservation across these species (**Figure 7**).

## 4 Discussion

In this study, the mitochondrial genome was initially assembled based on long-read data. This assembly strategy involved assembling all raw sequencing data first, followed by extracting sequences belonging to the mitochondrial genome. However, this approach had certain limitations. First, *de novo* assembly of the entire dataset consumed considerable computational resources, and most of the assembled contigs corresponded to nuclear genome sequences irrelevant to our research. Moreover, the assembly generated thousands of contigs, making the accurate identification of mitochondrial sequences labor-intensive and time-consuming. Additionally, screening became particularly challenging for highly complex mitochondrial genomes. Therefore, in future studies, the use of specialized tools for the preliminary enrichment of mitochondrial sequences could reduce the data volume for subsequent assembly, thereby saving computational resources and avoiding tedious and error-prone manual screening. Accordingly, it would be worthwhile to develop dedicated algorithms for the precise identification of long-reads with mitochondrial sequence characteristics from high-throughput sequencing data prior to assembly.

Repetitive sequences are identical or symmetrical DNA sequence fragments that frequently occur in plant genomes, occupy a large proportion of plant mitogenomes, and play important roles in genome size evolution, gene expression regulation, and responses to stress (Wendel et al., 2016). Repetitive DNA is generally classified into two major categories: tandem repeats and dispersed repeats. Tandem repeats consist of adjacent repeat units arranged in a head-to-tail manner, whereas dispersed repeats are distributed throughout the genome without physical adjacency. Simple sequence repeats (SSRs), a subclass of tandem repeats, typically contain repeat motifs ranging from 1 to 6 bp. Due to their codominant inheritance and high polymorphism, SSRs are widely used as molecular markers in plant genetic studies (Khera et al., 2015). In the study, 294 SSRs were identified in the mitogenome of *D. chinense*, which provided a valuable marker resource for evaluating germplasm diversity and conducting species identification in related taxa. Dispersed repeat sequences constitute a class of mobile DNA sequences capable of relocating within the genome, thereby gradually modifying functional genes and contributing to evolutionary processes (Bennetzen and Wang, 2014). These elements can modulate gene expression and influence phenotypic traits in plants (Lisch, 2013). In the present study, 1,054 dispersed repeat pairs were identified in the mitogenome of *D. chinense* mitogenome. Although their precise biological functions remain unclear, these repeats are likely to play important roles in shaping mitogenome structure and driving evolutionary dynamics.

Mitochondrial plastid DNA refers to DNA fragments that have migrated from the plastid genome to the mitochondrial genome, belonging to a type of horizontal gene transfer (HGT), and it is one of the main drivers of land plant evolution (Prasad et al., 2022). In this investigation, 17 homologous fragments were detected between the chloroplast and mitochondrial genomes of *D. chinense*, which occupied 1.31% of the entire mitochondrial genome. nine PCGs (*ndhJ*, *petG*, *petL*, *psbE*, *psbF*, *psbJ*, *psbL*, *rpl*20, *rpoC1*) were found to migrate from the plastome to the mitogenome in *D. chinense*. In angiosperms, it is common for tRNA genes to transfer from the plastome to the mitogenome (Bi et al., 2019). This phenomenon was also detected in the organellar genomes of *D. chinense*, including the tRNA genes *trnH-GUG*, *trnI-CAU*, *trnM-CAU*, *trnN-GUU*, *trnP-UGG*, and *trnW-CCA*. (**Supplementary Table 6**). In addition to the 15 complete genes mentioned above, some of the transferred PCGs and the tRNA genes were incomplete, and this phenomenon had also been observed in other species (Jiang et al., 2023). Whether these genes were still functional was an open question.

Codon usage bias analysis offers insights into the evolutionary adaptation and functional conservation of plant mitochondrial genomes. Despite substantial variations in genome size, structural arrangement and nucleotide composition among plant species, the core mitochondrial-encoded proteins remain highly conserved during evolution (Li et al., 2011b). In the present study, codon usage preference analysis revealed that leucine (Leu), serine (Ser), and arginine (Arg) were the predominant amino acids, while methionine (Met) and tryptophan (Trp) presented lower abundance. This conserved distribution pattern was consistent with most angiosperm species (Ma et al., 2022), indicating that the amino acid usage bias of plant mitogenomes was subject to strong evolutionary constraint. Such preference may also optimize mitochondrial gene translation efficiency and maintain the stable operation of energy metabolism pathways, reflecting adaptive characteristics during the long-term evolution of woody plants.

In this study, *D. chinense* and *D. racemosum* clustered together within the order Saxifragales, and the overall phylogenetic structure based on mitochondrial DNA sequences agreed well with the APG classification framework. Structural variation in plant mitogenomes is one of their most prominent features, with significant differences often observed even within the same genus or species. The *D. chinense* mitogenome assembled in this study exhibited a complex multi-branched, multi-chromosomal conformation. Specifically, it was formed by six interconnected contigs with a total length of approximately 916.5 kb, a structure that could not be readily resolved into classical circular DNA molecules. In contrast, the published mitogenome of its congener, *D. racemosum*, also comprised over ten contig sequences. By connecting certain contigs, the authors ultimately reconstructed a structure consisting of a “large circular chromosome (834.4 kb) and a small linear fragment (69.8 kb).” Similar structural features had also been reported in the tea plant (*Camellia sinensis* cv. ‘Zhuyeqi’) (Chen et al., 2025). The mitogenome of this tea cultivar consisted of one circular chromosome and six linear chromosomes, with a total length of 911,255 bp. Furthermore, a comparable hybrid configuration was documented in the halophyte *Halogeton glomeratus* (Wang et al., 2025), whose mitogenome comprised two circular chromosomes (168,414 bp and 144,793 bp, respectively) and one linear structure (19,991 bp). These structural configurations indicated that the mitogenomes of some species could not be fully characterized by a single simple circular DNA molecule. These findings suggested that plant mitogenomes might possess even greater structural diversity than previously anticipated. Comparative synteny analysis revealed a high degree of collinearity between the mitogenomes of *D. chinense* and *D. racemosum*, as evidenced by numerous conserved syntenic blocks. However, substantial genomic rearrangements were also detected between the two species, indicating dynamic structural variation despite overall syntenic conservation.

RNA editing was a widespread post-transcriptional process in the mitochondria of higher plants and constituted a key step in mitochondrial gene expression. This modification could give rise to initiation and termination codons that were not present in the original genomic DNA sequence. In general, RNA editing–mediated codon modifications enhance the conservation of encoded proteins, thereby increasing sequence similarity with homologous proteins across species and facilitating efficient mitochondrial gene expression. In addition, nonsynonymous substitutions induced by RNA editing may result in substantial functional alterations in mitochondrial genes, particularly through the generation of novel start and stop codons (Ichinose and Sugita, 2017; Hao et al., 2021). For instance, RNA editing was reported to convert ACG (Thr) into AUG (Met), thereby establishing potential transcription start sites (Zandueta-Criado and Bock, 2004). Similarly, RNA editing was also found to produce premature stop codons, which could result in truncated protein products. Notably, in this study, eight editing sites were identified that had the capacity to generate start or stop codons: *cox*1-2 (Thr to Met), *cox*2-443 (Thr to Met), *cox*2-698 (Thr to Met), *nad*1-2 (Thr to Met), *nad4L*-2 (Thr to Met), *nad*5-782 (Thr to Met), *nad*5-875 (Thr to Met), *nad*7-224 (Thr to Met), *rps*1-590 (Thr to Met), *rps*10-62 (Thr toMet), *rps*3-1364 (Thr to Met), *ccmFC*-1315 (Arg to End), and *rps*10-391 (Arg to End). The exact roles of these factors during plant growth and development required further comprehensive exploration.

## 5 Conclusions

In summary, the complete mitochondrial genome of *D. chinense* was assembled using a hybrid sequencing strategy integrating MGISEQ-2000 and Nanopore platforms. The mitogenome exhibited a complex multibranched structure, with a total length of 916,504 bp, and contained 39 PCGs, 19 tRNA genes, and 3 rRNA genes. We detected 17 homologous fragments between the mitochondrial and chloroplast genomes, with a combined length of 11,981 bp, representing 1.31% of the mitogenome. RNA editing analysis indicated that most editing events in protein-coding genes were cytidine-to-uridine conversions. Phylogenetic analysis suggested a close relationship between *D. chinense* and *D. racemosum*. Collectively, this study provided a high-quality mitogenome resource for *D. chinense* and established a solid foundation for future investigations into intracellular gene transfer, mitochondrial genome evolution, and functional genomics in this species.

## Data Availability

The data supporting this study are publicly available. The mitogenome sequence (Genbank: contig1 PZ247528, contig2 PZ247529) and chloroplast genome sequence (Genbank: PZ247530) have been deposited in the GenBank database (https://www.ncbi.nlm.nih.gov/). These accession numbers are also provided in the article
